# Case study of treatment of metatarsus adductus with Jones boot modification and convenient photocopy technology for monitoring progress

**DOI:** 10.1186/1757-1146-4-S1-P35

**Published:** 2011-05-20

**Authors:** Sylvia McAra, Chamali Egodagamage, Lachlan Newcombe, Dana Standring

**Affiliations:** 1 School of Community Health Charles Sturt University, Albury, NSW, 2641, Australia

## 

A 13 month old female child attended the CSU Allied Health Clinic student training podiatry service for assessment and treatment of her pronounced bilateral metatarsus adductus. Her deformity was mobile and a grade 4 on the right foot and grade 3 on the left using Bleck’s classification scale.  On presentation, each foot was able to be corrected to a straight lateral border with moderate hand pressure. As such she was assessed as suitable for Jones boot modification therapy. Her foot alignment was captured by standing her on the office photocopier with support from held hands. Manipulations and Jones boot modifications were carried out. She wore the boots for most daily activities and 2 pairs were used to accommodate her foot growth over this period. She was reviewed monthly and progressed to almost complete resolution over the period of 1 year. Her foot alignment was monitored at monthly reviews with the photocopier method. This method provides excellent snapshots of functional alignment and was highly convenient for the practitioners. The child was very compliant with this “game” and this was in contrast to attempts at X-ray, which was not procurable due to her noncompliance. A series of progressive photocopies with excellent black/white contrast outline the borders of both feet and illustrate the progress of this case. The Jones Boot modification technique is also illustrated with photographs in this poster presentation.

